# Supplementary material for: Pharmacological Inhibition of WIP1 Sensitizes Acute Myeloid Leukemia Cells to the MDM2 Inhibitor Nutlin-3a
Source: Biomedicines. 2021 Apr 6;9(4):388. doi: 10.3390/biomedicines9040388 (PMC8067413; doi:10.3390/biomedicines9040388)
Supplement: Supplementary file 1 [file biomedicines-09-00388-s001.pdf]

Supplementary Material

Supplementary Tables

Table S1. Combination index analysis of TP53-wt AML cells

TP53-wt AML cell lines	Drug concentration [µM]		24h			48h			72h		
			**WIP1i**								
			**5**	**10**	**20**	**5**	**10**	**20**	**5**	**10**	**20**
**MOLM-13**	**Nut-3a**	**0.5**	0.24	0.25	0.33	0.79	0.83	0.85	0.84	0.85	0.88
		**1**	0.35	0.47	0.60	0.90	0.92	0.93	0.93	0.94	0.95
		**2.5**	0.72	0.70	0.77	0.95	0.96	0.97	0.96	0.98	0.98
		**5**	0.81	0.79	0.84	0.99	0.99	0.99	0.98	0.98	0.98
**MV-4-11**		**0.5**	0.23	0.33	0.29	0.22	0.18	0.16	0.44	0.54	0.60
		**1**	0.24	0.26	0.29	0.25	0.24	0.19	0.70	0.80	0.88
		**2.5**	0.41	0.51	0.48	0.39	0.47	0.30	1.28	1.36	1.50
		**5**	0.29	0.63	0.67	0.45	0.45	0.45	2.11	2.17	2.30
**OCI-AML3**		**0.5**	0.06	0.08	0.33	0.30	0.25	0.66	0.87	0.77	4.88
		**1**	0.90	0.09	0.11	0.26	0.25	0.28	0.91	1.90	3.21
		**2.5**	0.11	0.15	0.09	0.38	0.35	0.31	0.86	1.25	0.92
		**5**	0.10	0.08	0.04	0.34	0.34	0.31	0.79	0.98	0.89

Table S2. Characteristics of primary AML samples

Sample ID	Specimen Type	Karyotype	*FLT3*	*NPM1*	*TP53*
#1	PB	*NA*	ITD	mut	wt
#2	BM	48, XY, +6, +15 [30]	wt	wt	wt
#3	PB	46 XY [20]	ITD	mut	wt
#4	BM	*NA*	wt	wt	wt
#5	BM	46 XX [15]	wt	wt	wt
#6	BM	46, XY [20]	ITD	mut	wt
#7	BM	46, XY [20]	ITD	mut	wt
#8	BM	46, XY, del(2)(p21), t(5;11)(q23;q24) [20]	TKD	wt	wt
#9	BM	46, XX [15]	ITD	mut	wt
#10	BM	Complex	ITD	wt	wt
#11	BM	Complex	wt	wt	mut
#12	PB	Complex	wt	wt	mut
#13	BM	*NA*	wt	wt	mut

BM: bone marrow; ITD: internal tandem duplication; mut: mutated; NA: not available; PB: peripheral blood; TKD: mutated in the tyrosine kinase domain; wt: wildtype

Table S3. Top 20 upregulated (red) and downregulated (blue) genes in MV-4-11 cells treated with Nut-3a+WIP1i *vs*. control

Gene	Description	Chromosome	Fold change	p value
MDM2	MDM2 Proto-Oncogene, E3 Ubiquitin Protein Ligase	chr12	17.03	0.003
TP53INP1	Tumor Protein P53 Inducible Nuclear Protein 1	chr8	5.70	0.008
XPC	Xeroderma Pigmentosum, Complementation Group C	chr3	4.86	0.019
ZMAT3	Zinc Finger, Matrin-Type 3	chr3	4.66	0.001
CDKN1A	Cyclin-Dependent Kinase Inhibitor 1A (P21, Cip1)	chr6	3.68	0.038
TOM1L1	Target Of Myb1 Like 1 Membrane Trafficking Protein	chr17	2.87	0.014
DYRK1B	Dual Specificity Tyrosine-(Y)-Phosphorylation Regulated Kinase 1B	chr19	2.69	0.036
MEI4	Meiotic Double-Stranded Break Formation Protein 4	chr6	2.53	0.001
SUMO4	Small Ubiquitin-Like Modifier 4	chr6	2.43	0.045
B3GAT1	Beta-1,3-Glucuronyltransferase 1	chr11	2.41	0.024
COL2A1	Collagen, Type II, Alpha 1	chr12	2.35	0.003
IRAK1BP1	Interleukin 1 Receptor Associated Kinase 1 Binding Protein 1	chr6	2.35	0.015
ALDH1A2	Aldehyde Dehydrogenase 1 Family, Member A2	chr15	2.20	0.025
ITGAD	Integrin Alpha D	chr16	2.13	0.032
HLA-A	Major Histocompatibility Complex, Class I, A	chr6	2.11	0.048
PROM1	Prominin 1	chr4	2.10	0.015
SIM1	Single-Minded Family Bhlh Transcription Factor 1	chr6	2.08	0.014
NAPSA	Napsin A Aspartic Peptidase	chr19	2.07	0.011
CYSRT1	Cysteine-Rich Tail Protein 1	chr9	2.07	0.039
NBR1	Neighbor Of BRCA1 Gene 1	chr17	2.03	0.020
HSPA14	Heat Shock 70kda Protein 14	chr10	0.28	0.002
UNG	Uracil DNA Glycosylase	chr12	0.30	0.039
RGSL1	Regulator Of G-Protein Signaling Like 1	chr1	0.35	0.038
FRMD3	FERM Domain Containing 3	chr9	0.35	0.002
ABHD17C	Abhydrolase Domain Containing 17C	chr15	0.35	0.018
TIMELESS	Timeless Circadian Clock	chr12	0.36	0.042
BCAT1	Branched Chain Amino-Acid Transaminase 1, Cytosolic	chr12	0.36	0.014
ARPC1A	Actin Related Protein 2/3 Complex Subunit 1A	chr7	0.36	0.003
PSMG4	Proteasome (Prosome, Macropain) Assembly Chaperone 4	chr6	0.37	0.021
RRM2	Ribonucleotide Reductase M2	chr2	0.37	0.049
HELLS	Helicase, Lymphoid-Specific	chr10	0.39	0.027
LBR	Lamin B Receptor	chr1	0.40	0.031
GSTA2	Glutathione S-Transferase Alpha 2	chr6	0.40	0.025
SLC27A2	Solute Carrier Family 27 (Fatty Acid Transporter), Member 2	chr15	0.41	0.018

ICAM3	Intercellular Adhesion Molecule 3	chr19	0.42	0.006
DCUN1D5	DCN1, Defective In Cullin Neddylation 1, Domain Containing 5	chr11	0.42	0.016
SETD4	SET Domain Containing 4	chr21	0.42	0.043
SETD7	SET Domain Containing (Lysine Methyltransferase) 7	chr4	0.43	0.008
TREML2	Triggering Receptor Expressed On Myeloid Cells-Like 2	chr6	0.43	0.032

Table S4. Top 20 upregulated (red) and downregulated (blue) genes in NOMO-1 cells treated with Nut3a+WIP1i *vs*. control

Gene	Description	Chromosome	Fold change	p value
FABP4	Fatty Acid Binding Protein 4, Adipocyte	chr8	45.25	0.003
CD36	Cd36 Molecule (Thrombospondin Receptor)	chr7	36.76	0.016
LACC1	Laccase (Multicopper Oxidoreductase) Domain 1	chr13	20.39	0.015
SLC39A8	Solute Carrier Family 39 (Zinc Transporter), Member 8	chr4	14.83	0.033
TNFRSF9	Tumor Necrosis Factor Receptor Superfamily, Member 9	chr1	8.28	0.003
ME1	Malic Enzyme 1, Nadp(+)-Dependent, Cytosolic	chr6	5.98	0.015
PLIN2	Perilipin 2	chr9	5.98	0.019
CCL18	Chemokine (C-C Motif) Ligand 18	chr17	5.24	0.024
IER3	Immediate Early Response 3	chr6	5.21	0.038
G0S2	G0/G1 Switch 2	chr1	4.89	0.006
GPR84	G Protein-Coupled Receptor 84	chr12	4.50	0.005
LPAR6	Lysophosphatidic Acid Receptor 6	chr13	4.23	0.012
ZFP36L1	Zfp36 Ring Finger Protein-Like 1	chr14	3.97	0.020
IL7R	Interleukin 7 Receptor	chr5	3.94	0.042
PHLDA1	Pleckstrin Homology-Like Domain, Family A, Member 1	chr12	3.92	0.007
SOD2	Superoxide Dismutase 2, Mitochondrial	chr6	3.92	0.042
PDK4	Pyruvate Dehydrogenase Kinase, Isozyme 4	chr7	3.86	0.044
ALDH1L2	Aldehyde Dehydrogenase 1 Family, Member L2	chr12	3.81	0.008
LDHAL6B	Lactate Dehydrogenase A-Like 6b	chr15	3.34	0.022
TRPV5	Transient Receptor Potential Cation Channel Subfamily V, Member 5	chr7	3.10	0.033
STK25	Serine/Threonine Kinase 25	chr2	0.37	0.022
NEIL3	Nei-Like Dna Glycosylase 3	chr4	0.37	0.041
CDCA5	Cell Division Cycle Associated 5	chr11	0.37	0.039
TMEM55B	Transmembrane Protein 55b	chr14	0.37	≤0.001
DUS2	Dihydrouridine Synthase 2	chr16	0.37	0.010
PYCR1	Pyrroline-5-Carboxylate Reductase 1	chr17	0.37	0.034
LASP1	Lim And Sh3 Protein 1	chr17	0.38	0.042
PAK1	P21 Protein (Cdc42/Rac)-Activated Kinase 1	chr11	0.38	0.047
DEFB1	Defensin, Beta 1	chr8	0.38	0.023
PPP2R5D	Protein Phosphatase 2, Regulatory Subunit B, Delta	chr6	0.38	0.025
INPP5D	Inositol Polyphosphate-5-Phosphatase D	chr12	0.38	0.020

ZNF692	Zinc Finger Protein 692	chr1	0.38	0.040
HHIP	Hedgehog Interacting Protein	chr4	0.38	0.029
DTYMK	Deoxythymidylate Kinase	chr2	0.38	0.009
UBXN11	Ubx Domain Protein 11	chr1	0.38	0.050
PBX2	Pre-B-Cell Leukemia Homeobox 2	chr6	0.38	0.006
CDCA2	Cell Division Cycle Associated 2	chr8	0.38	0.039
MAP4K2	Mitogen-Activated Protein Kinase Kinase Kinase Kinase 2	chr11	0.38	0.016
ANXA8	Annexin A8	chr10	0.39	0.019
ANAPC4	Anaphase Promoting Complex Subunit 4	chr4	0.39	0.017

Table S5. Top 20 upregulated (red) and downregulated (blue) genes in MV-4-11 cells treated with Nut3a+WIP1i *vs.* WIP1i

Gene	Description	Chromosome	Fold change	p value
MDM2	MDM2 Proto-Oncogene, E3 Ubiquitin Protein Ligase	chr12	17.03	0.002
TP53INP1	Tumor Protein P53 Inducible Nuclear Protein 1	chr8	5.70	0.008
XPC	Xeroderma Pigmentosum, Complementation Group C	chr3	4.86	0.019
ZMAT3	Zinc Finger, Matrin-Type 3	chr3	4.66	≤0.001
CDKN1A	Cyclin-Dependent Kinase Inhibitor 1A (P21, Cip1)	chr6	3.68	0.038
TOM1L1	Target Of Myb1 Like 1 Membrane Trafficking Protein	chr17	2.87	0.013
DYRK1B	Dual Specificity Tyrosine-(Y)-Phosphorylation Regulated Kinase 1B	chr19	2.69	0.036
MEI4	Meiotic Double-Stranded Break Formation Protein 4	chr6	2.53	≤0.001
SUMO4	Small Ubiquitin-Like Modifier 4	chr6	2.43	0.045
B3GAT1	Beta-1,3-Glucuronyltransferase 1	chr11	2.41	0.024
COL2A1	Collagen, Type II, Alpha 1	chr12	2.35	0.003
IRAK1BP1	Interleukin 1 Receptor Associated Kinase 1 Binding Protein 1	chr6	2.35	0.015
ALDH1A2	Aldehyde Dehydrogenase 1 Family, Member A2	chr15	2.20	0.025
ITGAD	Integrin Alpha D	chr16	2.13	0.031
HLA-A	Major Histocompatibility Complex, Class I, A	chr6	2.11	0.047
PROM1	Prominin 1	chr4	2.10	0.014
SIM1	Single-Minded Family Bhlh Transcription Factor 1	chr6	2.08	0.014
NAPSA	Napsin A Aspartic Peptidase	chr19	2.07	0.010
CYSRT1	Cysteine-Rich Tail Protein 1	chr9	2.07	0.039
NBR1	Neighbor Of BRCA1 Gene 1	chr17	2.03	0.019
HSPA14	Heat Shock 70kda Protein 14	chr10	0.28	0.001
UNG	Uracil DNA Glycosylase	chr12	0.30	0.038
RGSL1	Regulator Of G-Protein Signaling Like 1	chr1	0.35	0.038
FRMD3	FERM Domain Containing 3	chr9	0.35	0.001
ABHD17C	Abhydrolase Domain Containing 17C	chr15	0.35	0.018
TIMELESS	Timeless Circadian Clock	chr12	0.36	0.041
BCAT1	Branched Chain Amino-Acid Transaminase 1, Cytosolic	chr12	0.36	0.014
ARPC1A	Actin Related Protein 2/3 Complex Subunit 1A	chr7	0.36	0.003
PSMG4	Proteasome (Prosome, Macropain) Assembly Chaperone 4	chr6	0.37	0.020
RRM2	Ribonucleotide Reductase M2	chr2	0.37	0.048
HELLS	Helicase, Lymphoid-Specific	chr10	0.39	0.026

LBR	Lamin B Receptor	chr1	0.40	0.030
GSTA2	Glutathione S-Transferase Alpha 2	chr6	0.40	0.025
SLC27A2	Solute Carrier Family 27 (Fatty Acid Transporter), Member 2	chr15	0.41	0.017
ICAM3	Intercellular Adhesion Molecule 3	chr19	0.42	0.006
DCUN1D5	DCN1, Defective In Cullin Neddylation 1, Domain Containing 5	chr11	0.42	0.016
SETD4	SET Domain Containing 4	chr21	0.42	0.043
SETD7	SET Domain Containing (Lysine Methyltransferase) 7	chr4	0.43	0.007
TREML2	Triggering Receptor Expressed On Myeloid Cells-Like 2	chr6	0.43	0.031

Table S6. Top 20 upregulated (red) and downregulated (blue) genes in NOMO-1 cells treated with Nut3a+WIP1i *vs.* WIP1i

Gene	Description	Chromosome	Fold change	p value
CCL3	Chemokine (C-C Motif) Ligand 3	chr17	13.93	0.032
LACC1	Laccase (Multicopper Oxidoreductase) Domain Containing 1	chr13	11.00	0.024
TNFRSF9	Tumor Necrosis Factor Receptor Superfamily, Member 9	chr1	8.40	0.002
GPR84	G Protein-Coupled Receptor 84	chr12	8.34	0.015
G0S2	G0/G1 Switch 2	chr1	5.35	0.003
RGL1	Ral Guanine Nucleotide Dissociation Stimulator-Like 1	chr1	5.31	0.026
SOD2	Superoxide Dismutase 2, Mitochondrial	chr6	4.29	0.033
RASGRP1	Ras Guanyl Releasing Protein 1	chr15	3.97	0.038
ZFP36L1	Zfp36 Ring Finger Protein-Like 1	chr14	3.61	0.003
CALCOCO1	Calcium Binding And Coiled-Coil Domain 1	chr12	3.32	0.003
ARL4C	Adp-Ribosylation Factor Like Gtpase 4c	chr2	3.14	0.043
ME1	Malic Enzyme 1, Nadp(+)-Dependent, Cytosolic	chr6	3.01	0.036
DUSP16	Dual Specificity Phosphatase 16	chr12	2.99	0.014
ADGRE1	Adhesion G Protein-Coupled Receptor E1	chr19	2.99	0.022
GPR132	G Protein-Coupled Receptor 132	chr14	2.71	0.029
PLA2G7	Phospholipase A2, Group Vii	chr6	2.66	0.028
TNFSF15	Tumor Necrosis Factor (Ligand) Superfamily, Member 15	chr9	2.64	0.021
FABP6	Fatty Acid Binding Protein 6, Ileal	chr5	2.64	0.009
NFAT5	Nuclear Factor Of Activated T-Cells 5, Tonicity-Responsive	chr16	2.60	0.049
COX7A1	Cytochrome C Oxidase Subunit Viia Polypeptide 1	chr19	2.60	0.044
ADGRA3	Adhesion G Protein-Coupled Receptor A3	chr4	0.17	0.049
KCNQ5	Potassium Channel, Voltage Gated Kqt-Like Subfamily Q, Member 5	chr6	0.17	0.039
MARC1	Mitochondrial Amidoxime Reducing Component 1	chr1	0.20	0.036
CEP70	Centrosomal Protein 70kda	chr3	0.21	0.042
CA2	Carbonic Anhydrase Ii	chr8	0.22	0.018
SGTA	Small Glutamine-Rich Tetratricopeptide Repeat (Tpr)-Containing, Alpha	chr19	0.24	0.045
BCAP29	B-Cell Receptor-Associated Protein 29	chr7	0.24	0.023
SDHAF3	Succinate Dehydrogenase Complex Assembly Factor 3	chr7	0.25	0.008
IKBKAP	Inhibitor Of Kappa Light Polypeptide Gene Enhancer In B-Cells, Kinase Complex-Associated Protein	chr9	0.26	0.025
CR1	Complement Component (3b/4b) Receptor 1	chr1	0.30	0.041
DTD1	D-Tyrosyl-Trna Deacylase 1	chr20	0.30	0.038

FANCG	Fanconi Anemia Complementation Group G	chr9	0.30	0.001
HIST2H4A; HIST2H4B	Histone Cluster 2, H4a; Histone Cluster 2, H4b	chr1	0.30	0.011
PELP1	Proline, Glutamate And Leucine Rich Protein 1	chr17	0.31	0.021
NCK2	Nck Adaptor Protein 2	chr2	0.32	0.038
HIST2H4B; HIST2H4A	Histone Cluster 2, H4b; Histone Cluster 2, H4a	chr1	0.32	0.025
MRPL14	Mitochondrial Ribosomal Protein L14	chr6	0.33	0.045
COLGALT1	Collagen Beta(1-O)Galactosyltransferase 1	chr19	0.33	0.032
HELLS	Helicase, Lymphoid-Specific	chr10	0.33	0.047
TNSF13B	Tumor Necrosis Factor (ligand) Superfamily, Member 13B	chr13	0.33	0.048

Table S7. Top 20 upregulated (red) and downregulated (blue) genes in MV-4-11 cells treated with Nut3a+WIP1i *vs.* Nut-3a

**Gene**	**Description**	**Chromosome**	**Fold change**	**p value**
MDM2	MDM2 Proto-Oncogene, E3 Ubiquitin Protein Ligase	chr12	6.77	0.005
MMP16	Matrix Metallopeptidase 16 (Membrane-Inserted)	chr8	3.27	0.042
SETBP1	SET Binding Protein 1	chr18	2.91	0.023
ZMAT3	Zinc Finger, Matrin-Type 3	chr3	2.69	0.002
ZBTB33	Zinc Finger And BTB Domain Containing 33	chrX	2.60	0.017
TMEM229B	Transmembrane Protein 229B	chr14	2.39	0.001
TIGAR	TP53 Induced Glycolysis Regulatory Phosphatase	chr12	2.33	0.049
COL2A1	Collagen, Type II, Alpha 1	chr12	2.30	≤0.001
GPA33	Glycoprotein A33 (Transmembrane)	chr1	2.27	0.001
VSIG10	V-Set And Immunoglobulin Domain Containing 10	chr12	2.27	0.014
ZFP90	ZFP90 Zinc Finger Protein	chr16	2.25	0.044
HERC6	HECT And RLD Domain Containing E3 Ubiquitin Protein Ligase Family Member 6	chr4	2.23	0.021
ZNF91	Zinc Finger Protein 91	chr19	2.23	0.006
CCDC188	Coiled-Coil Domain Containing 188	chr22	2.20	0.043
ZNF260	Zinc Finger Protein 260	chr2	2.17	0.013
FAM19A2	Family With Sequence Similarity 19 (Chemokine (C-C Motif)-Like), Member A2	chr17	2.14	0.004
HMX2	H6 Family Homeobox 2	chr15	2.14	0.017
ISG20	Interferon Stimulated Exonuclease Gene 20kda	chr19	2.14	0.028
ZNF404	Zinc Finger Protein 404	chr12	2.14	0.002
YPEL1	Yippee Like 1	chr10	2.13	0.011
FOXM1	Forkhead Box M1	chr4	0.33	0.043
YWHAH	Tyrosine 3-Monooxygenase/Tryptophan 5-Monooxygenase Activation Protein, Eta	chr16	0.34	0.021
VAT1	Vesicle Amine Transport 1	chr1	0.36	0.041
AARS	Alanyl-Trna Synthetase	chr5	0.36	0.023
RARA	Retinoic Acid Receptor, Alpha	chr1	0.38	0.024
ORC1	Origin Recognition Complex Subunit 1	chr19	0.38	0.008
RNF145	Ring Finger Protein 145	chr12	0.39	0.043
RNF41	Ring Finger Protein 41, E3 Ubiquitin Protein Ligase	chr16	0.40	0.012
MCM6	Minichromosome Maintenance Complex Component 6	chr17	0.40	0.016

SPTBN1	Spectrin, Beta, Non-Erythrocytic 1	chr11	0.40	0.025
DNAAF1	Dynein, Axonemal, Assembly Factor 1	chr1	0.40	0.034
E2F8	E2F Transcription Factor 8	chr12	0.41	0.047
RNASE2	Ribonuclease, Rnase A Family, 2 (Liver, Eosinophil-Derived Neurotoxin)	chr17	0.43	0.022
SENP3	SUMO1/Sentrin/SMT3 Specific Peptidase 3	chr11	0.43	0.050
SMG6	SMG6 Nonsense Mediated Mrna Decay Factor	chr21	0.43	0.038
CDCA5	Cell Division Cycle Associated 5	chr8	0.44	0.028
CHAF1B	Chromatin Assembly Factor 1, Subunit B (P60)	chr9	0.44	0.029
CHRNE	Cholinergic Receptor, Nicotinic Epsilon	chr3	0.44	0.021
PLD1	Phospholipase D1, Phosphatidylcholine-Specific	chr15	0.44	0.037
USP17L2	Ubiquitin Specific Peptidase 17-Like Family Member 2	chr11	0.44	0.035

Table S8. Top 20 upregulated (red) and downregulated (blue) genes in NOMO-1 cells treated with Nut3a+WIP1i *vs.* Nut-3a

**Gene**	**Description**	**Chromosome**	**Fold change**	**p value**
FABP4	Fatty Acid Binding Protein 4, Adipocyte	chr8	38.59	0.004
CALCOCO1	Calcium Binding And Coiled-Coil Domain 1	chr12	4.82	0.015
ZNF518B	Zinc Finger Protein 518B	chr4	4.53	0.019
PHGDH	Phosphoglycerate Dehydrogenase	chr1	4.47	0.050
LY6G6C	Lymphocyte Antigen 6 Complex, Locus G6C	chr6	4.06	0.050
ME1	Malic Enzyme 1, NADP(+)-Dependent, Cytosolic	chr6	3.61	0.024
ANXA5	Annexin A5	chr4	3.12	0.042
FAM219A	Family With Sequence Similarity 219, Member A	chr9	3.05	0.013
KCNAB1	Potassium Channel, Voltage Gated Subfamily A Regulatory Beta Subunit 1	chr3	3.03	0.036
IL23R	Interleukin 23 Receptor	chr1	2.95	0.042
STAC2	SH3 And Cysteine Rich Domain 2	chr17	2.83	0.019
C17orf99	Chromosome 17 Open Reading Frame 99	chr17	2.71	0.049
SMG5	SMG5 Nonsense Mediated Mrna Decay Factor	chr1	2.69	0.024
HEATR4	HEAT Repeat Containing 4	chr14	2.69	0.046
KMO	Kynurenine 3-Monooxygenase (Kynurenine 3-Hydroxylase)	chr1	2.68	0.043
UGT2B10	UDP Glucuronosyltransferase 2 Family, Polypeptide B10	chr4	2.68	0.034
TNFRSF9	Tumor Necrosis Factor Receptor Superfamily, Member 9	chr1	2.62	0.028
TNFSF4	Tumor Necrosis Factor (Ligand) Superfamily, Member 4	chr1	2.53	0.001
IGDCC3	Immunoglobulin Superfamily, DCC Subclass, Member 3	chr15	2.53	0.049
PRMT8	Protein Arginine Methyltransferase 8	chr12	2.51	0.017
PPP1R27	Protein Phosphatase 1, Regulatory Subunit 27	chr17	0.08	0.048
HIST2H4B; HIST2H4A	Histone Cluster 2, H4b; Histone Cluster 2, H4a	chr1	0.08	0.033
CTSG	Cathepsin G	chr14	0.09	0.016
IL18RAP	Interleukin 18 Receptor Accessory Protein	chr2	0.12	0.016
HIST1H3B	Histone Cluster 1, H3b	chr6	0.15	0.048
SPP1	Secreted Phosphoprotein 1	chr4	0.16	0.042
HIST1H2AK	Histone Cluster 1, H2ak	chr6	0.17	0.012
PDAP1	PDGFA Associated Protein 1	chr7	0.17	0.045
HIST1H2BI	Histone Cluster 1, H2bi	chr6	0.18	0.041

HIST2H3A	Histone Cluster 2, H3a	chr1	0.18	0.012
ATP5I	ATP Synthase, H+ Transporting, Mitochondrial Fo Complex Subunit E	chr4	0.20	0.036
SLC6A8	Solute Carrier Family 6, Member 8	chrX	0.21	0.018
CLEC11A	C-Type Lectin Domain Family 11, Member A	chr19	0.21	0.018
RPS27A	Ribosomal Protein S27a	chr2	0.21	0.043
CCR2	Chemokine (C-C Motif) Receptor 2	chr3	0.21	0.016
INPP5K	Inositol Polyphosphate-5-Phosphatase K	chr17	0.22	0.046
PSMD8	Proteasome 26S Subunit, Non-Atpase 8	chr19	0.22	0.036
ART3	ADP-Ribosyltransferase 3	chr4	0.23	0.011
HIST1H2AE	Histone Cluster 1, H2ae	chr6	0.23	0.048
DEPDC1	DEP Domain Containing 1	chr1	0.24	0.049

Table S9. P53 target* and signature** genes that are significantly upregulated in MV-4-11 or NOMO-1 cells treated with Nut-3a+WIP1i *vs*. control.

Gene	Description	Chromosome	Fold change	p value	Cell line
MDM2	MDM2 proto-oncogene, E3 ubiquitin protein ligase	chr12	40.50	0.002	MV-4-11
CDKN1A	cyclin-dependent kinase inhibitor 1A (p21, Cip1)	chr6	12.73	0.012	MV-4-11
TP53INP1	tumor protein p53 inducible nuclear protein 1	chr8	12.73	0.006	MV-4-11
EDA2R	ectodysplasin A2 receptor	chrX	11.47	0.021	MV-4-11
TIGAR	TP53 induced glycolysis regulatory phosphatase	chr12	10.78	0.001	MV-4-11
TRIM22	UTR5 best transcript NM_001199573	chr11	10.48	0.004	MV-4-11
XPC	xeroderma pigmentosum, complementation group C	chr3	9.85	0.006	MV-4-11
APOBEC3H	apolipoprotein B mRNA editing enzyme, catalytic polypeptide-like 3H	chr22	7.94	0.038	MV-4-11
SESN1	sestrin 1	chr6	6.96	0.000	MV-4-11
ZMAT3	zinc finger, matrin-type 3	chr3	6.87	0.001	MV-4-11
POLH	polymerase (DNA directed), eta	chr6	5.82	0.025	MV-4-11
FDXR	ferredoxin reductase	chr17	5.06	0.006	MV-4-11
MLF2	myeloid leukemia factor 2	chr12	4.79	0.000	MV-4-11
PLK2	polo-like kinase 2	chr5	4.23	0.003	MV-4-11
FBXO22	F-box protein 22	chr15	4.11	0.031	MV-4-11
CYFIP2	cytoplasmic FMR1 interacting protein 2	chr5	3.92	0.034	MV-4-11
ANXA4	intronic best transcript NM_001153	chr2	3.53	0.032	MV-4-11
DCP1B	decapping mRNA 1B	chr12	3.16	0.038	MV-4-11
LAPTM5	lysosomal protein transmembrane 5	chr1	3.12	0.046	MV-4-11
DDB2	damage-specific DNA binding protein 2	chr11	2.97	0.024	MV-4-11
RETSAT	retinol saturase (all-trans-retinol 13,14-reductase)	chr2	2.83	0.039	MV-4-11
TNFSF9	tumor necrosis factor (ligand) superfamily, member 9	chr19	2.83	0.003	MV-4-11

MRPL49	mitochondrial ribosomal protein L49	chr11	2.79	0.017	MV-4-11
DOCK8	dedicator of cytokinesis 8	chr9	2.77	0.037	MV-4-11
TNFRSF10C	tumor necrosis factor receptor superfamily, member 10c, decoy without an intracellular domain	chr8	2.55	0.002	MV-4-11
FBXO32	F-box protein 32	chr8	2.36	0.036	MV-4-11
ISCU	iron-sulfur cluster assembly enzyme	chr12	2.36	0.011	MV-4-11
MR1	major histocompatibility complex, class I-related	chr1	2.25	0.012	MV-4-11
IER5	immediate early response 5	chr1	2.17	0.013	MV-4-11
ZNF195	zinc finger protein 195	chr11	2.13	0.021	MV-4-11
BTBD10	BTB (POZ) domain containing 10	chr11	2.10	0.021	MV-4-11
HHAT	hedgehog acyltransferase		2.07	0.010	MV-4-11
GADD45A	growth arrest and DNA-damage-inducible, alpha	chr1	2.06	0.013	MV-4-11
NINJ1	ninjurin 1	chr9	2.17	0.012	NOMO-1
LACC1	laccase (multicopper oxidoreductase) domain containing 1	chr13	20.39	0.015	NOMO-1
VWCE	von Willebrand factor C and EGF domains	chr11	2.10	0.007	NOMO-1

* p53 targets from Fischer M, *Oncogene* 2017.
** genes from the "Hallmark p53 pathway" MSigDB signature.

Supplementary Figures

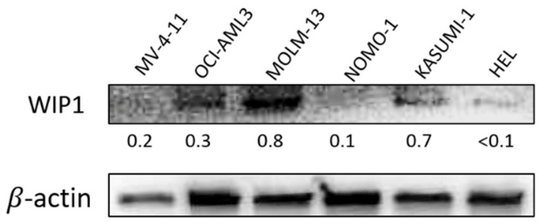

Figure S1. WIP1 protein expression in AML cell lines. Representative western blot of WIP1 expression in *TP53*-wt (MV-4-11, OCI-AML3, MOLM-13) and *TP53*-mut (NOMO-1, KASUMI-1, HEL) cell lines. Values indicate WIP1 expression normalized on β-actin.

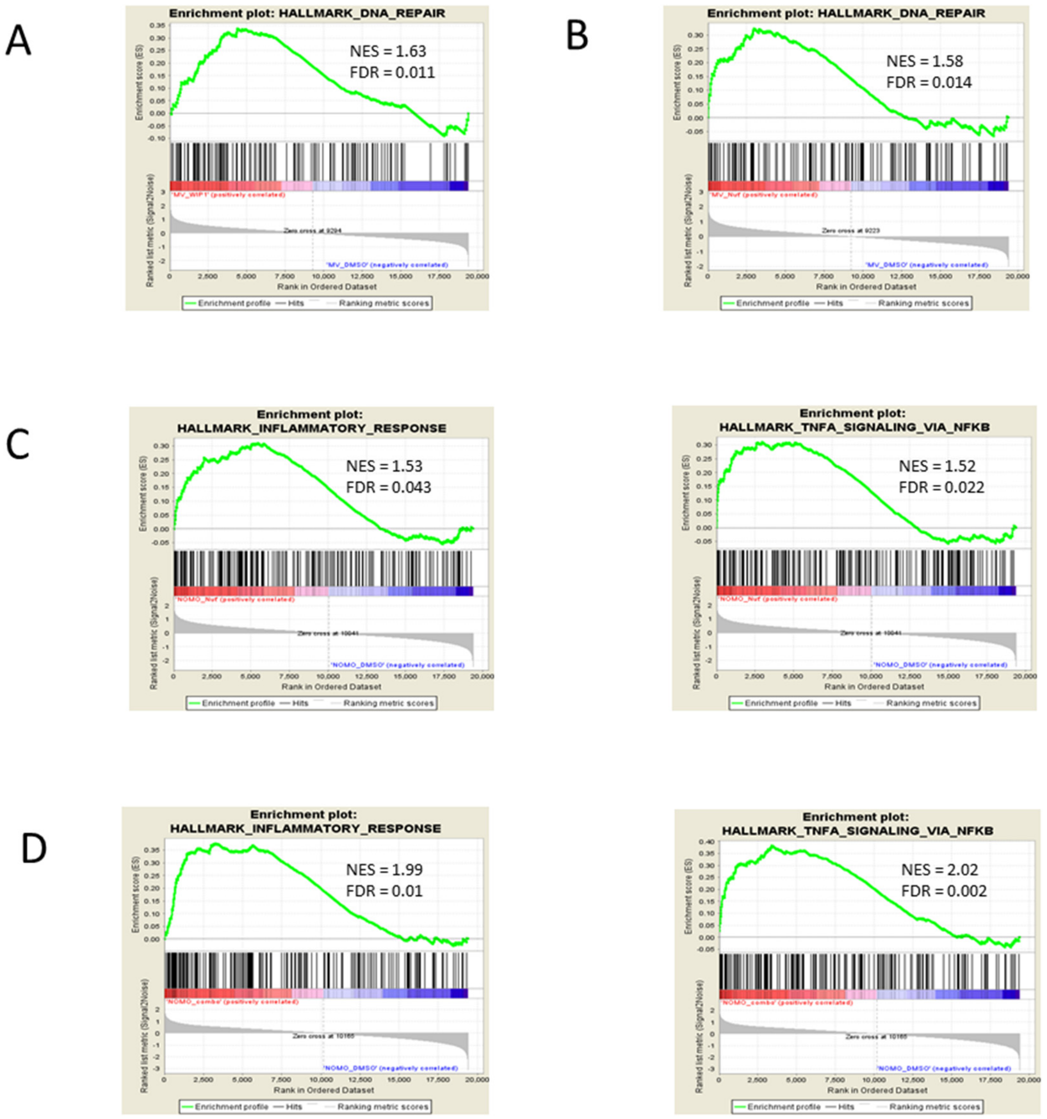

Figure S2. Additional transcriptional signatures in single and combined-treatment enriched in MV-4-11 and NOMO-1 cells. GSEA plots of MV-4-11 cells treated with (**A**) WIP1i *vs*. DMSO, (**B**) Nut-3a *vs*. DMSO. GSEA common plots of NOMO-1 cells treated with (**C**) Nut-3a *vs*. DMSO, (**D**) Nut-3a+WIP1i *vs*. DMSO.

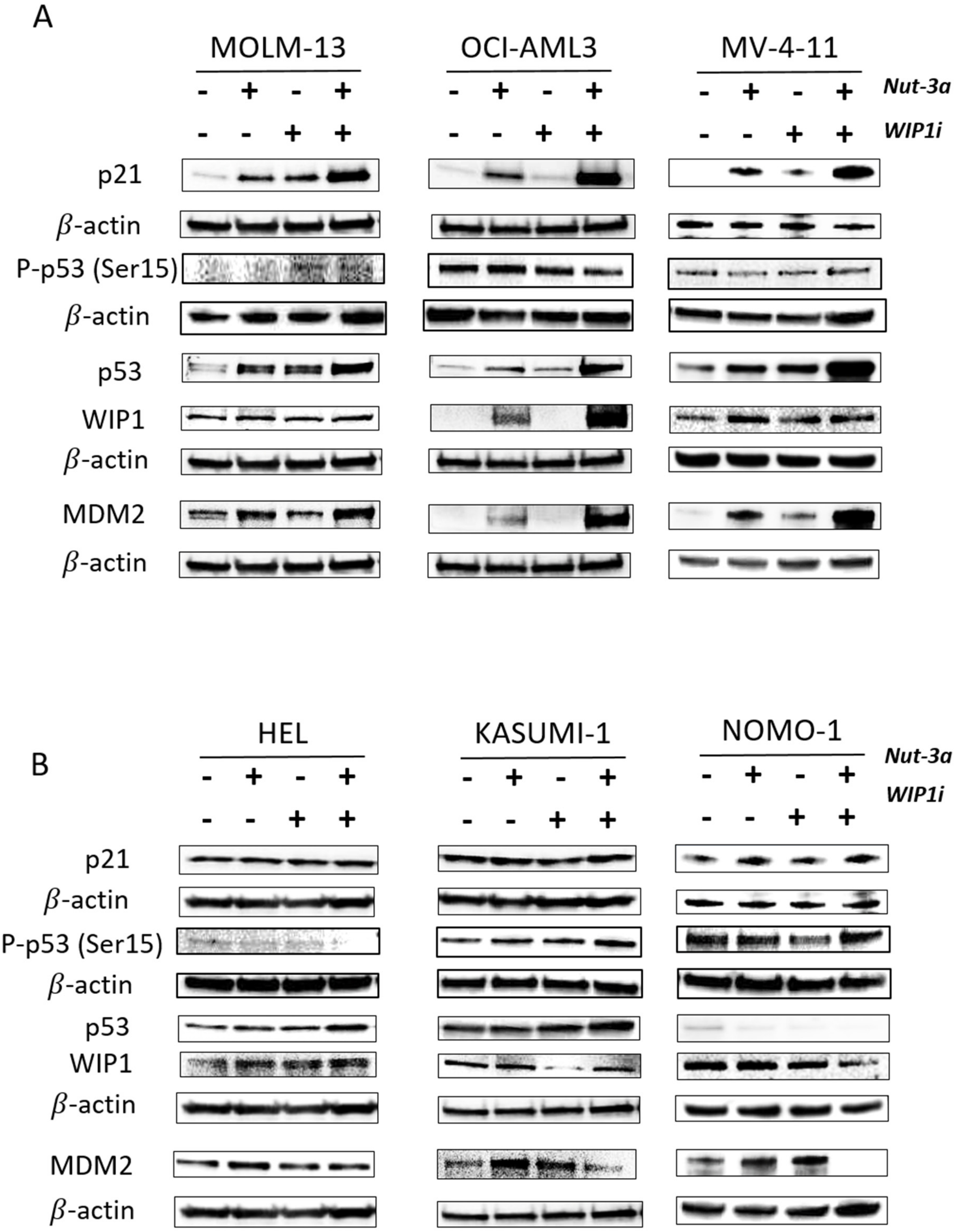

Figure S3. Expression changes of p53 and its related genes induced by the treatment in *TP53*-wt and *TP53*-mut cells. Representative western blots of p53-related genes in (A) MOLM-13, OCI-AML3 and MV-4-11 and (B) HEL, KASUMI-1 and NOMO-1 cells after 16h of treatment (Nut-3a 0.5, 2.5 and 0.5 µM; WIP1i 10, 20, 5 µM respectively for TP53-wt cells; Nut-3a 5 µM and WIP1i 20 µM for all the *TP53*-mut cells).
